# Preferences for life-sustaining treatments and advance directives among heart failure patients: a systematic review and meta-analysis

**DOI:** 10.1186/s12872-026-05945-z

**Published:** 2026-05-25

**Authors:** Shiva Khaleghparast, Saeideh Mazloomzadeh, Aydin Feyzi, Fahimeh Farrokhzadeh, Sanaz Sadeghi, Amirhossein Ghaseminejad-Raeini, Sara Adimi, Amirali Soheili, Samira Chaibakhsh

**Affiliations:** 1 Cardiovascular Nursing Research Center, Rajaie Cardiovascular Institute, Tehran, Iran; 2 School of Nursing and Midwifery, Shahid Beheshti University of Medical Sciences, Tehran, Iran; 3 Cardiovascular Research Center, Rajaie Cardiovascular Institute, Tehran, Iran; 4 York University, Toronto, Canada; 5 Cardiovascular Epidemiology Research Center, Rajaie Cardiovascular Institute, Tehran, Iran

**Keywords:** Heart failure, Life-sustaining treatments, Preference, Advance directives

## Abstract

**Background:**

Advance directives (ADs) play a fundamental role in guiding end-of-life (EOL) care, yet patient preferences regarding surrogate decision-making, life-sustaining treatments (LSTs), and cardiopulmonary resuscitation (CPR) fluctuate extensively. Understanding these preferences is crucial for person-centered and culturally sensitive advance care planning (ACP), particularly heart failure patients.

**Methods:**

A systematic review and meta-analysis were conducted to evaluate patient preferences regarding ADs. Databases including PubMed, Scopus, Web of Science, and Embase were searched for studies published up to March 6, 2024 that reported on preferences related to surrogate decision-making, LSTs, or CPR in patients with heart failure. Data was pooled using a random-effects model. Sensitivity analyses were conducted to assess the influence of outlier studies. Risk of publication bias was evaluated using Begg’s and Egger’s tests.

**Results:**

Thirteen studies involving varied international populations were included. The pooled estimate of patients preferring surrogate decision-making was 53.66% (95% CI: 16.9%–90.43%), increasing to 71% (95% CI: 51.64%–91.34%) after excluding an outlier. Preference for receiving LSTs was 48.8% (95% CI: 37.84%–59.77%), increasing to 52.28% (95% CI: 35.95%–59.88%) after exclusion of one study. The pooled proportion of patients preferring to refuse CPR was 43.32% (95% CI: 29.13%–57.52%). Cultural factors, health status, and prior communication influenced these preferences. No significant publication bias was identified.

**Conclusion:**

Preferences regarding ADs among patients with heart failure are diverse and influenced by cultural setting, disease morbidity, and prior ACP discussions. A substantial proportion of patients prefer surrogate decision-making and limited life-prolonging interventions. These findings highlight the need for culturally sensitive, personalized ACP that accommodates developing patient values and decision-making roles over time.

## Introduction

An advance directive (AD) is a legal document enabling individuals to express their preferences regarding medical treatments in conditions they become incapable of making decisions themselves [1, 2] .ADs are essential in preserving patient autonomy, particularly during serious illness or incapacitation, by guiding healthcare workers and family members to respect the patient’s wishes [3, 4]. They often cover a range of medical interventions, including decisions about life-sustaining treatments (LST), thus playing a key role in end-of-life (EOL) care planning [5, 6].

Heart failure represents a clinically meaningful context for advance-directive preferences study, because patients experience recurrent episodes of decompensation, high symptom burden, and unpredictable illness trajectories. These features create repeated decision points regarding LSTs, making HF a distinct and clinically important population for examining EOL preferences. EOL decisions involve complicated considerations about commencing, withholding, or withdrawing LSTs such as cardiopulmonary resuscitation (CPR), intubation, extracorporeal membrane oxygenation (ECMO), and dialysis [7–9]. While these interventions can extend patients’ life, they might also prolong suffering or diminish the quality of life, especially in patients with end-stage diseases [10]. The application of ADs helps ensure that EOL care are in accordance with patient values and preferences, facilitating clearer communication between patients, families, and healthcare providers [11–13].

In patients with heart failure (HF), the necessity for ADs is particularly well-defined, due to the disease’s chronic and progressive clinical course [12, 14]. HF patients often experience unpredictable health declines and might need vital choices regarding LSTs like CPR or dialysis [9, 15, 16]. Early discussions about ADs and the designation of a surrogate decision-maker are essential to navigate these challenges and to maintain patient-centered care [17, 18]. Despite the critical role of AD in ensuring patient-centered EOL care, many individuals with HF still lack documented ADs. This lack of preparation can lead to significant emotional distress for families and may result in medical interventions that are misaligned with the patient’s personal values and preferences. In a 2012 study conducted in the United States [19], only 41.0% of HF patients had an AD at the time of enrollment. While the majority of these documents designated a surrogate decision-maker (90.4%), fewer than half included specific instructions regarding LSTs such as cardiopulmonary resuscitation or mechanical ventilation—underscoring the urgent need to enhance advance care planning (ACP) in this population [19].

Furthermore, low, with only a few having prior personal experience related to a parent’s or friend’s health care decisions [20]. These findings collectively emphasize a concerning gap in both documentation and awareness of ACP among HF patients, pointing to the necessity for further research in field of AD.

The aim of this systematic review is to explore the ADs and preferences of HF patients regarding LSTs and end-of-life decisions by gathering relevant former studies in field of AD among HF patients. After a systematic search on AD among HF patients and a meta-analysis on relevant studies, we seek to report different preferences of these patients regarding LST, EOL, and surrogate decision making.

## Method

### Study design and ethical considerations

This review was carried out following the guidelines of the Preferred Reporting Items for Systematic Reviews and Meta-Analyses (PRISMA) [21], and received ethical approval from the Ethics Committee of Rajaei Cardiovascular Institute (IR.RHC.REC.1403.064). Considering the alignment of the research question with the study objectives through a systematic review method, this research was a systematic review conducted in 2024, including articles related to HF patients’ preferences regarding EOL decisions and ADs from the beginning until March 6, 2024.

### Search strategy

Four databases (Web of Sciences, Embase, Scopus, PubMed) were used for the systematic search. To prevent the loss of other articles, the Google Scholar database was also used to complete the search by checking five initial output pages. Keywords utilized for systematic search were: heart failure, patient preferences, and advance directive. Their related keywords were also completed by using MeSH Terms of Pubmed and EMTREE of Embase; such as: EOL decision-making, Advance care planning (ACP). Keywords were also completed by two specialists in the medical field, and the search with the final search query in databases was conducted by a library specialist. Detailed search query is available on Supplementary-1.

### Eligibility criteria

Studies were eligible for inclusion if they were original research articles with accessible full texts, published in any language. Eligible studies specifically addressed EOL decision-making, ADs, or patient preferences in individuals diagnosed with heart failure (HF). Studies were excluded if they were presented as posters, conference abstracts, presentations, letters to the editor, case reports, case series, or review articles. Additionally, studies were excluded if they lacked a clear definition of study outcomes, were qualitative in design, or if full-text access was not available through any means. Studies that did not report the relevant variables specifically for the HF subgroup were also excluded.

Only quantitative studies were included because the primary objective of this review was to generate pooled prevalence estimates. Qualitative studies were excluded because they do not provide extractable numerical data suitable for meta-analysis, and their inclusion would have introduced methodological heterogeneity that precludes statistical pooling.

### Study selection

A librarian (FF), under the supervision of the authors, extracted articles and omitted duplicated records. Two independent reviewers (AS and AF) conducted the initial screening of all retrieved records by evaluating titles and abstracts. During this phase, studies were categorized into three groups: related, possibly related, and unrelated to the research topic. Subsequently, the full texts of all studies marked as “related” or “possibly related” were independently reviewed by the same two reviewers to assess eligibility based on the predefined inclusion and exclusion criteria. A third reviewer (SK) facilitated a consensus meeting during which all “possibly related” studies and any disagreements between the initial reviewers were discussed. Final decisions regarding inclusion or exclusion were made through consensus among the three reviewers.

### Data extraction

A standardized data extraction form was developed to collect relevant variables from the included studies. Before formal data collection, the form was pilot-tested on a sample of studies to ensure clarity, consistency, and completeness. Following this pilot phase, data extraction was independently performed by two reviewers.

The extracted variables included (Table 1):

- *Study characteristics*: first author, year of publication, country of study, and sample size;
- *Participant demographics*: mean age and standard deviation, percentage of male participants, education level, and marital status;
- *Clinical characteristics*: prevalence of diabetes and hypertension within the study sample;
- *End-of-life decision-making preferences*: percentage of participants who preferred their physician to make EOL decisions, and percentage who designated a surrogate decision-maker;
- *Life-sustaining treatment preferences*: percentage of participants who preferred to receive LSTs, and percentage who declined cardiopulmonary resuscitation (CPR), indicating a form of do-not-resuscitate (DNR) preference.

For studies reporting multiple types of LSTs (e.g., mechanical ventilation, dialysis, feeding tube), the highest reported percentage among those options was used to represent the overall preference for receiving LSTs in that study. This decision was made to avoid heterogeneity seen in different studies in reporting LSTs making their data extractable for pooled analysis.

**Table 1:** Overview of extracted variables from included studies in the systematic review and meta-analysis

Category	Variable	Description
Study Characteristics	Author	First author of the study
	Year	Year of publication
	Country	Country where the study was conducted
	Sample Size	Total number of participants
Participant Demographics	Age (Mean ± SD)	Mean age and standard deviation of participants
	Gender (% Male)	Percentage of male participants
	Education Level	Educational background of participants
	Marital Status	Proportion of participants who were married
Clinical Characteristics	Diabetes Prevalence (%)	Percentage of participants with diabetes
	Hypertension Prevalence (%)	Percentage of participants with hypertension
EOL Decision-Making	% Choosing Physician for EOL Decisions	Percentage of participants preferring their doctor to make EOL decisions
	% Choosing Surrogate for EOL Decisions	Percentage of participants choosing a surrogate decision-maker
LST Preferences	% Preferring Life-Sustaining Treatment (LST)	Highest percentage among any LST options reported
	% Refusing CPR (DNR)	Percentage of participants indicating they did not want CPR

To account for contextual variability, we extracted the exact phrasing used in each study to assess preferences for LSTs.

### Quality assessment process

The methodological quality of the included studies was assessed using appropriate tools based on study design. The AXIS tool was applied for cross-sectional studies, the Newcastle–Ottawa Scale (NOS) was used for cohort studies, and the Cochrane Risk of Bias 2 (ROB-2) tool was employed for randomized controlled trials (RCTs). Each study was independently evaluated by two reviewers (AS and AF), and any discrepancies in scoring were resolved through discussion or consultation with a third reviewer (SK). An overall quality judgment (e.g., low, moderate, or high risk of bias) was made for each study based on the outcomes of the respective assessment tool, and these ratings were used to guide interpretation during synthesis.

### Data synthesis and statistical analysis

Following data extraction, all included studies were systematically summarized, and their key findings were synthesized narratively to identify overarching themes and patterns related to advance directives, life-sustaining treatment (LST) preferences, and end-of-life decision-making among heart failure patients. In addition to narrative synthesis, where appropriate, quantitative data from comparable studies were pooled for meta-analysis. LSTs were analyzed as a composite category because several included studies reported aggregated LSTs preferences without distinguishing between specific interventions. To avoid excluding these studies and to preserve statistical power, we synthesized LSTs collectively.

Statistical analyses were carried out with STATA software (version 16, The Sage Group plc, Newcastle upon Tyne, England, UK). Heterogeneity was quantified using I², with values exceeding 50% indicating substantial heterogeneity. In such cases, a random-effects model was employed rather than a fixed-effects model. Forest plots and confidence intervals were presented to help interpret the findings. Because the number of included studies was small, funnel plot tests for publication bias could not be performed. The effect size was expressed as the proportion. A p-value < 0.05 was deemed statistically significant.

## Results

**Fig. 1:**
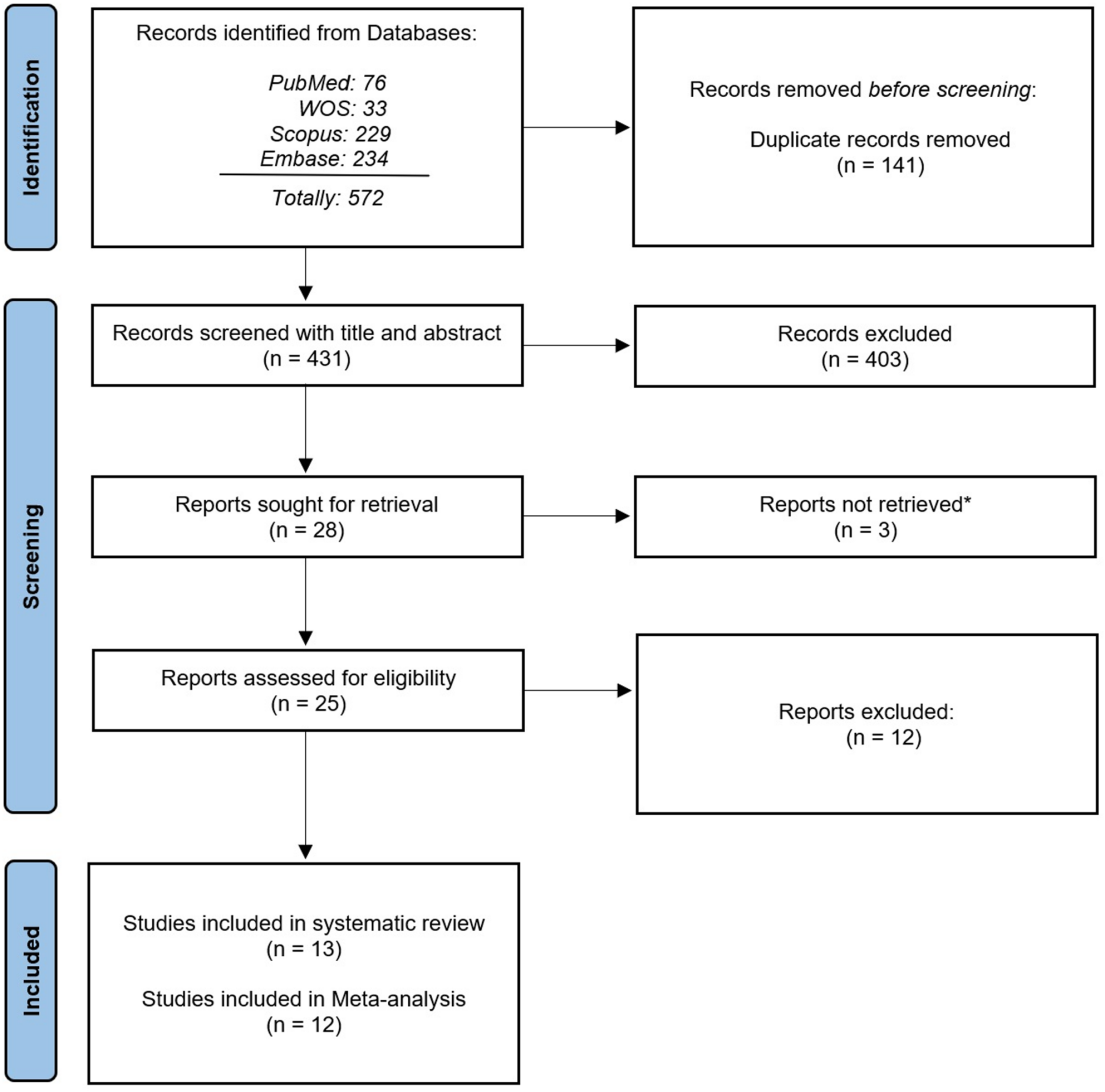
PRISMA 2020 flow diagram of study selection process for the systematic review and meta-analysis

A total of 572 records were initially identified through database searching, including 76 from PubMed, 33 from Web of Science, 229 from Scopus, and 234 from Embase. After removing 141 duplicate records, 431 unique records remained for screening based on titles and abstracts. Following the initial screening, 403 records were excluded for not meeting the inclusion criteria. The remaining 28 full-text reports were assessed for eligibility. Of these, 3 reports could not be retrieved, and 12 full-text articles were excluded for reasons such as irrelevant outcomes, population mismatch, or insufficient data reporting (Figure 1).

A total of 13 studies [4, 12, 13, 16, 19, 22–29] met the eligibility criteria and were included in the systematic review, of which 12 [4, 12, 16, 19, 22–29] were eligible for meta-analysis. The characteristics of the included studies are summarized in Table 2.The majority were observational studies, primarily employing a cross-sectional design. Most studies had a higher proportion of male participants. The studies were geographically diverse, with 4 conducted in the USA, 2 in Canada, 2 in Spain, 2 in South Korea, 1 in Switzerland, and 1 in Japan.

**Table 2:** Characteristics of all included studies for systematic review

Author (Year)	Country	Study Design	Sample Size (*n*)	Mean Age (SD)	% Male	Education Level	Setting	Marital Status	Diabetes (%)	Hypertension (%)	Main Outcomes	Note
Formiga (2004) [24]	Spain	Cross-sectional	80	79 (8.7)	42%	NR	Hospitalized elderly patients with HF	45% married	NR	NR	40% declined CPR; 66% preferred comfort care; 50% preferred home care; few had CPR discussions	Communication gaps; CPR rarely discussed; high preference for palliative care
Habal (2011)[25]	Canada	Cross-sectional	41	56.5 (10.6)	83%	NR	Outpatients at academic cardiology clinic	NR	NR	NR	76% unaware of ADs; 81% preferred CPR; 78% never discussed ACP with a physician	Major misconceptions; strong CPR preference despite poor awareness of ADs
Brunner-La Rocca (2012)[12]	Switzerland	Cross-sectional	622	76.9 (7.6)	59%	NR	Outpatients with chronic HF	58% married	36%	74%	33% had AD, 8% wanted CPR, 47% discussed EOL preferences	High awareness, low documentation of AD
Young (2017) [16]	USA	Cross-sectional	400	77.7 (12.5)	54%	47% above high school	Hospitalized or recent HF patients	54.5% married	45.5%	84.5%	40% had AD, 90% had surrogate, 46% had EOL discussion, 32% DNR	CPR preference linked to age, education; strong surrogate designation pattern
Kim (2020) [26]	South Korea	Cross-sectional	48	50.1 (0.94)	85.4%	47.9% above high school	Tertiary center HF patients	87.5% married	NR	NR	77.1% preferred aggressive LST; low awareness of AD law; spouse often preferred as surrogate	Cultural barriers and low legal knowledge impact planning
Kim (2021) [27]	South Korea	Cross-sectional	67	67 (11.8)	61.2%	NR	Hospital-based survey of advanced HF	64.2% married	NR	NR	32.8% had AD; 67.2% DNR preference; low discussion rate with medical staff	Discussions and support critical for AD completion
Trasancos-Escura (2021) [29]	Spain	Cross-sectional	32	81 (6.9)	53.1%	NR	Palliative HF patients (Spanish language)	53.1% married	NR	NR	68.8% refused CPR; 6.3% had AD; low awareness and major barriers to discussion	Study in Spanish; emphasizes need for patient/clinician education
Gordon (2017) [4]	USA	Cross-sectional	104	53 (14.3)	66%	NR	Urban, inner-city heart failure program	NR	NR	NR	26% aware of ADs; 33% DNR; 74% preferred CPR; 83% had surrogate decision-maker	High CPR preference despite DNR; mistrust and education gaps noted
Kitakata (2021) [28]	Japan	Cross-sectional	171	73 (9)	67.3%	NR	Outpatients with HF from multicenter cohort	NR	NR	NR	78.4% preferred CPR; 53.8% preferred LST; very low ACP discussion rate	Need for earlier ACP awareness and physician involvement
Campos (2022) [22]	Canada	Retrospective cohort	250	88.7 (7.9)	46.4%	NR	Deceased HF patients’ records in hospital	NR	21.6%	67.6%	32% had AD; 62% had DNR; many initially full-code changed to comfort-focused	Late shift in preferences; real-world variability in care planning
Dunlay (2012) [19]	USA	Prospective longitudinal cohort	249	79.8 (10.3)	48.6%	NR	Community-based HF cohort in Minnesota	NR	35.9%	92%	73% had AD; 99% had surrogate; only 25% had DNR among ADs	AD common; LST limitations infrequent despite surrogate assignment
El-Jawahri (2016) [23]	USA	Randomized Controlled Trial (RCT)	246	81 (8)	61%	NR	Hospitalized HF patients in palliative care intervention trial	NR	NR	NR	AD and surrogate designation improved with intervention; CPR preference dropped post-intervention	RCT showed positive impact of palliative intervention on ACP documentation and preferences; only baseline data used for analysis in this review
Cousino (2023) [13]	USA	Cross-sectional	Not applicable (dual cohort)	Not specified	NR	NR	Young adult HF patients (18–39 y) + caregivers	NR	NR	NR	High discordance between patient and caregiver preferences regarding LST and ADs	Distinct population (young adults); included only in narrative synthesis due to design

*NR* Not reported, *LST* Life sustaining treatment, *AD* advance directive, *HF* heart failure, *USA* United states of America

### Meta analysis

In total, 2,310 participants across 12 studies were included in the pooled analysis. Sample sizes ranged from 32 to 622 participants. The pooled mean age of participants was 76.6 years with a standard deviation (SD) of 12.7 years. On average, 60.3% of the participants were male. Marital status was reported in several studies, with approximately 61.6% of participants being married.

**Fig. 2:**
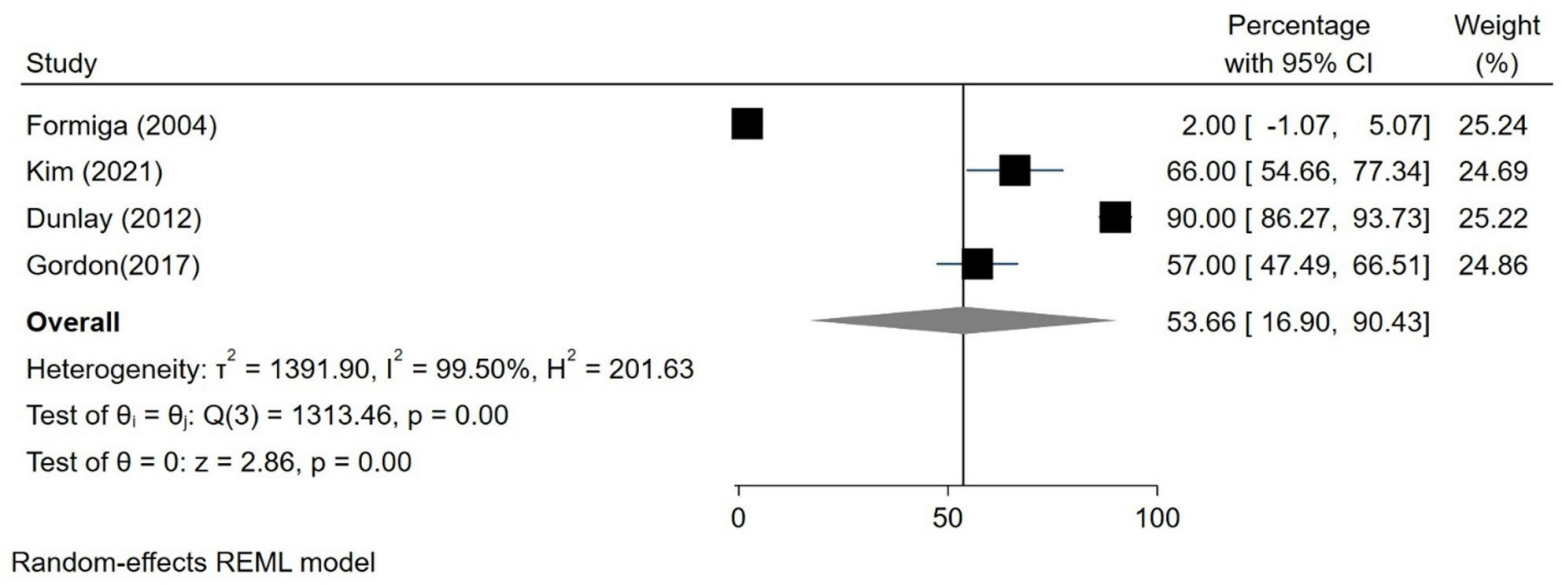
Pooled estimate of the proportion of individuals preferring surrogate decision-making

Only a subset of studies reported clinical characteristics: three studies reported diabetes prevalence with an average of 28.8%, and three reported hypertension prevalence averaging 70.8%. Four studies were analyzed to determine a pooled estimate of the percentage of people who preferred to have their surrogates make decisions for them. The pooled estimate was 53.66% (95% CI: 16.9% to 90.43%) (see Fig. 2). All reported percentages from these studies were above 57%, except for one study, which reported 2%.

**Fig. 3:**
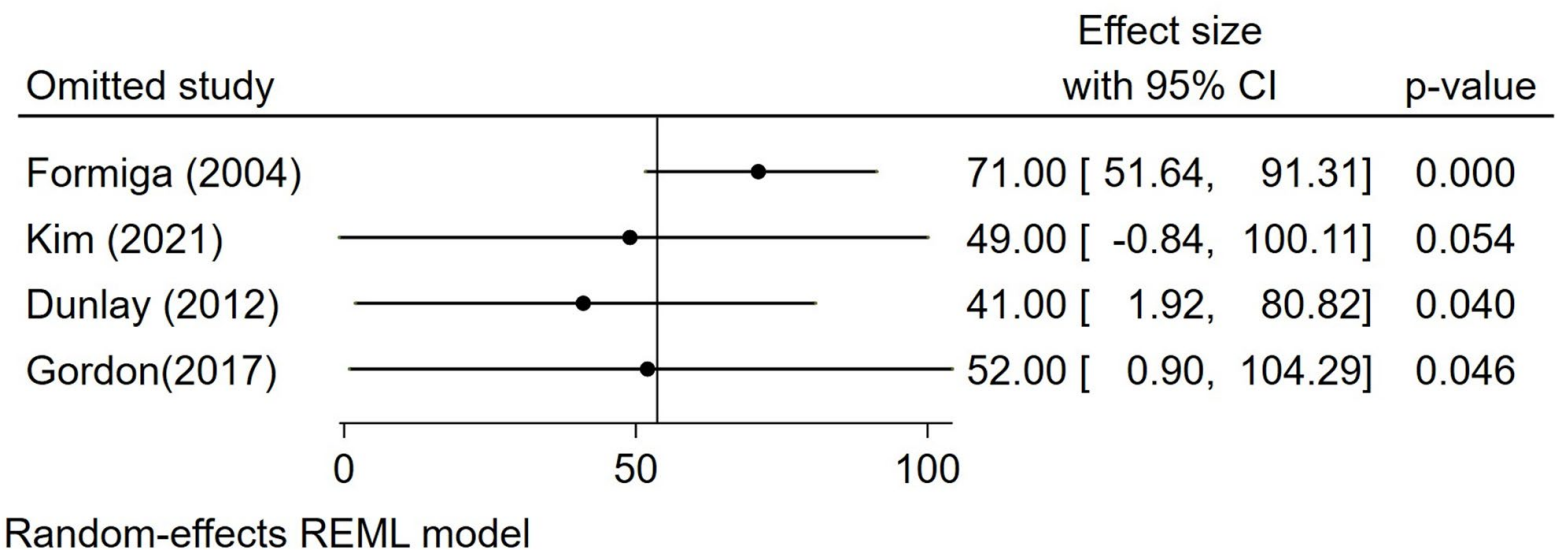
Sensitivity analysis: pooled estimate of the proportion of individuals preferring surrogate decision-making, excluding the outlier study (Formiga)

Sensitivity analysis was performed, and the results are shown in Fig. 3 By excluding the outlier (Formiga), the new pooled estimate increased to 71% (95% CI: 51.64% to 91.34%). Risk of bias was assessed using Begg and Egger tests, and both results indicated no significant risk of bias (p-values = 0.639 and 0.734, respectively).

**Fig. 4:**
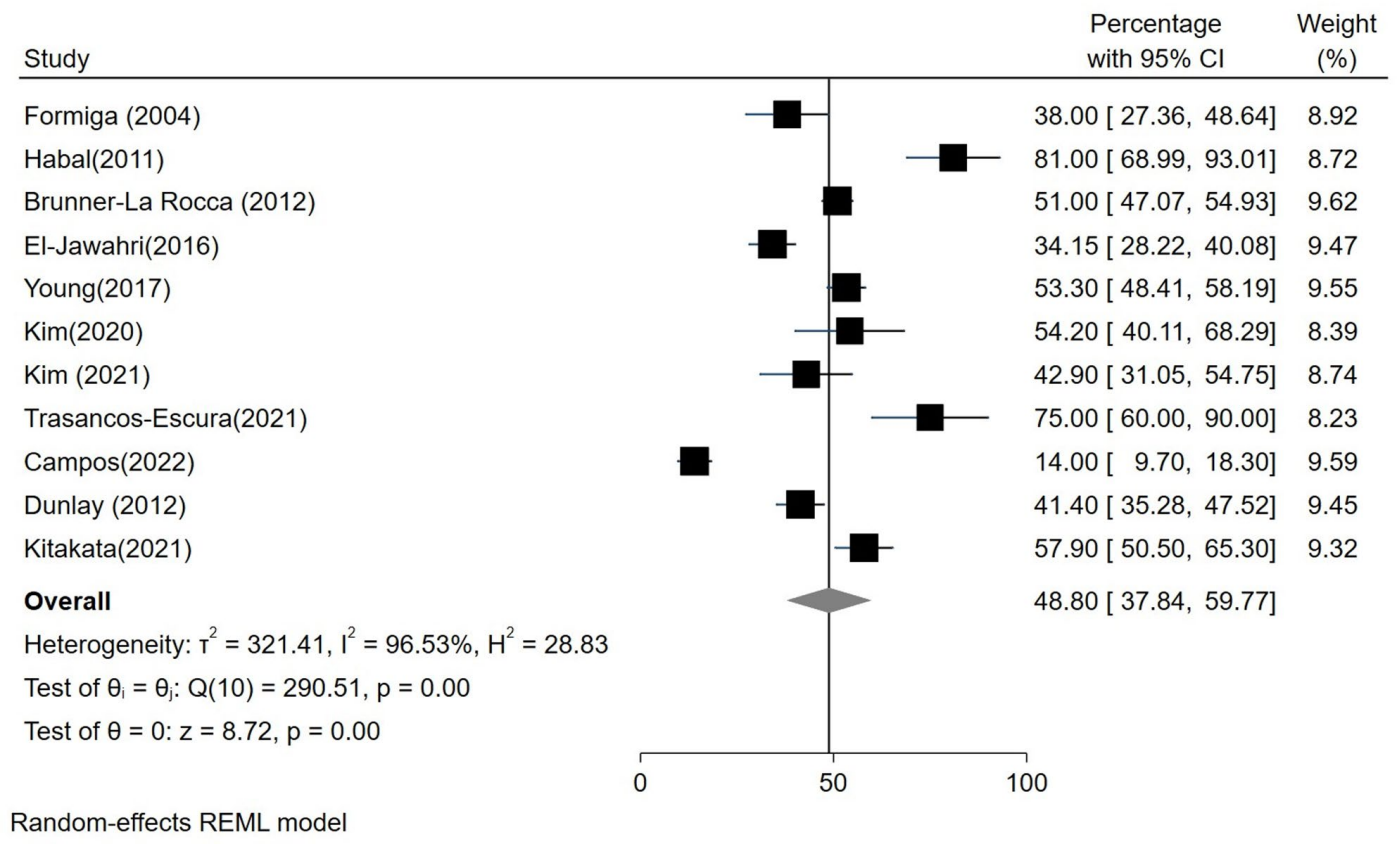
Pooled estimate of the proportion of individuals preferring life-sustaining treatments (LSTs)

Figure 4 including 10 studies illustrates the pooled estimates regarding preferences for LSTs [12, 16, 22–26, 29]. Habal et al. [25] reported the highest interest in using LSTs at 81%, while Campos et al. [22] reported the lowest interest at 14%. Overall, the meta-analysis indicated that the percentage of patients who preferred LSTs was 48.8%, with a 95% confidence interval (CI) of 37.84% to 59.77%.

**Fig. 5:**
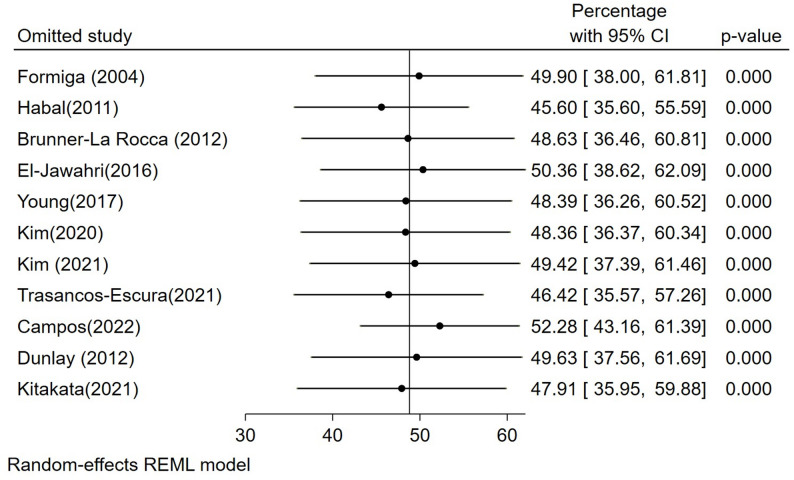
Sensitivity analysis: pooled estimate of the proportion of individuals preferring life-sustaining treatments (LSTs), excluding the outlier study (Campos et al.)

Sensitivity analysis was performed, and the results are shown in Fig. 5 after excluding Campos et al. [22] study which reported a lower outcome in comparison with other studies, and did not report extractable numerical data for the outcome of the interest, the new pooled estimate for preferences regarding LSTs was 52.28% with a 95%CI ranging from 35.95% to 59.88%. Risk of bias was assessed using Begg and Egger tests, and both results indicated no significant risk of bias (p-values = 0.426 and 0.436, respectively).

**Fig. 6:**
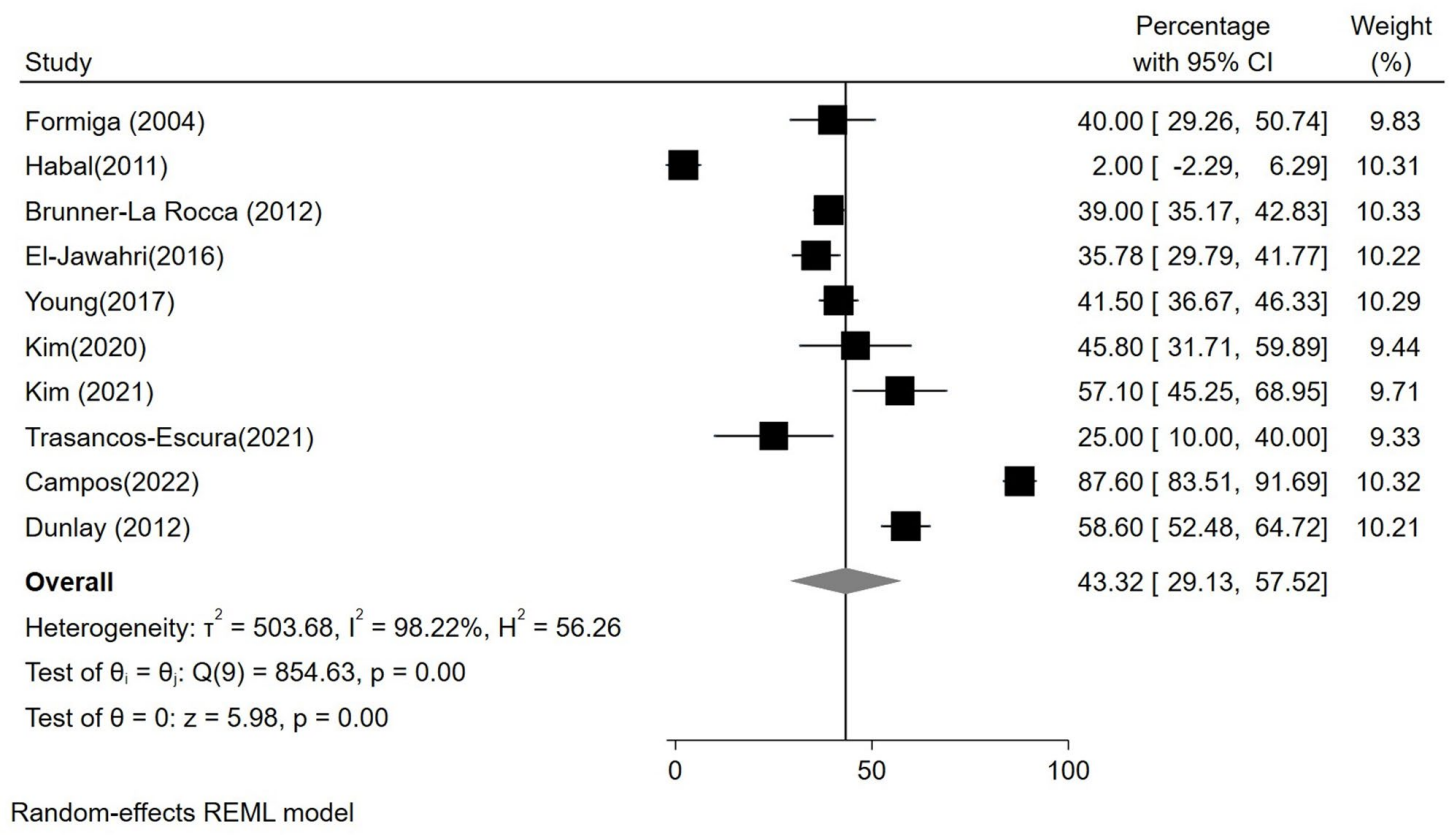
Pooled estimate of the proportion of individuals opposed to receiving cardiopulmonary resuscitation (CPR)

Ten studies were utilized to assess the percentage of individuals who did not wish to receive cardiopulmonary resuscitation (CPR) (Fig. 6) [12, 16, 22–27, 29, 30]. The highest and lowest percentages of participants opposed to the use of CPR were reported by Campos et al. (87.6%) [22] and Habal et al. (2%) [25], respectively. The pooled estimate indicated that, overall, the percentage of participants opposed to receiving CPR was 43.32%, with a 95% CI of 29.13 to 57.52.

**Fig. 7:**
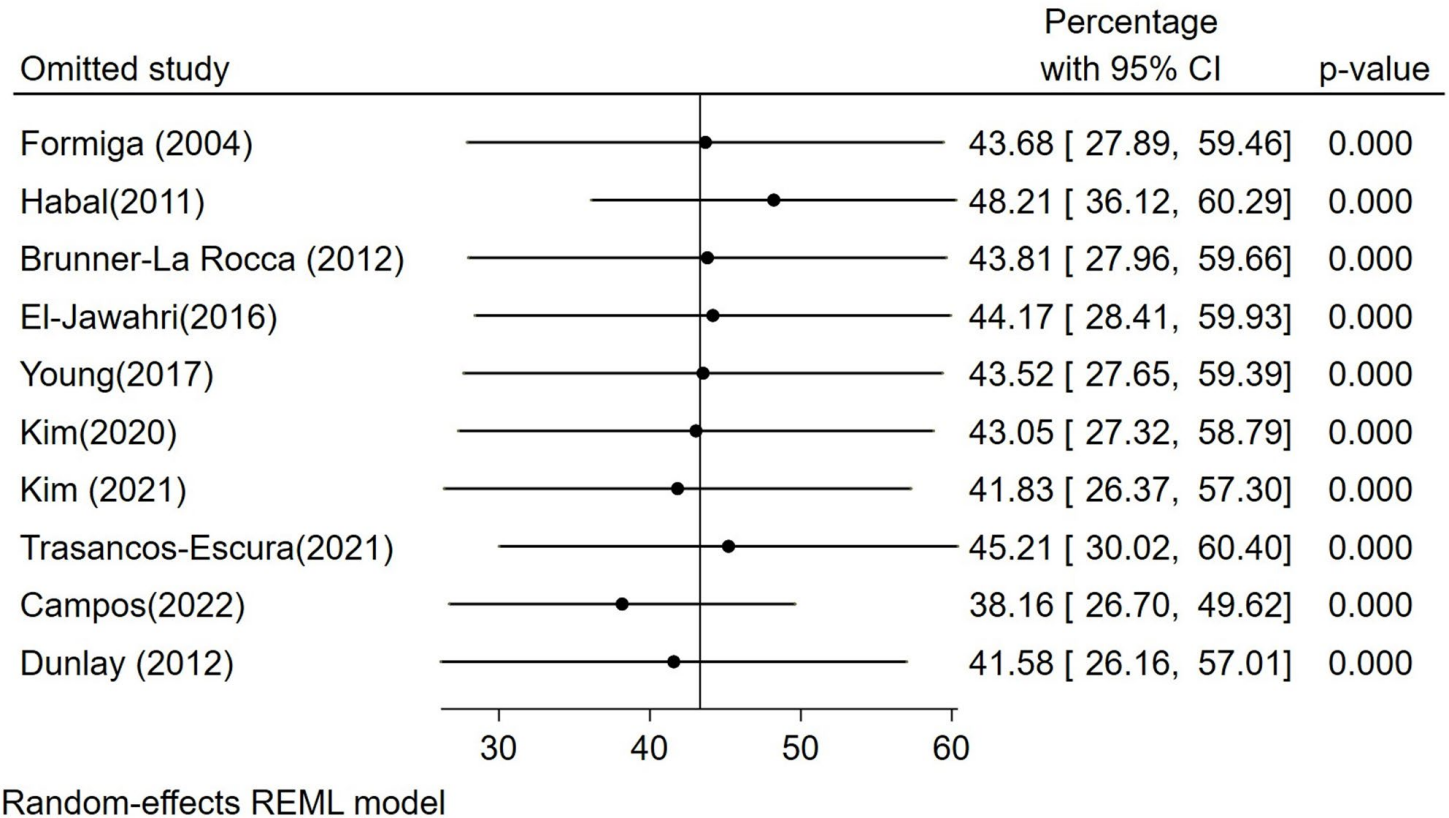
Sensitivity analysis: pooled estimates of individuals opposed to receiving cardiopulmonary resuscitation (CPR), excluding outlier studies

Sensitivity analysis was conducted, with results displayed in Fig. 7 Excluding the outlier (Campos et al.) decreased the new pooled estimate to 38.16% (95% CI: 26.7% to 49.62%). Removing the outlier (Habal et al.) resulted in a reduced pooled estimate of 48.21% (95% CI: 36.12% to 60.29%). Risk of bias was evaluated using Begg and Egger tests, both of which indicated no significant bias (p-values = 0.815 and 0.592, respectively).

### Quality assessment

A comprehensive quality assessment was conducted for all included studies based on their design type. For cross-sectional studies (*n* = 9) [4, 12, 16, 24–29], the AXIS tool was used, with most studies rated as “Good” quality (*n* = 7) [12, 16, 24, 26–29] and two rated as “Fair” [4, 25]. Cohort studies (n = 2) [19, 22] were evaluated using the Newcastle–Ottawa Scale (NOS), and both were judged to be of strong quality, scoring 7 out of 9. The single trial [23] was assessed using the ROB-2 tool and was judged to have “some concerns”, mainly due to lack of blinding and moderate follow-up loss, though it was otherwise well-designed and clearly reported.

## Discussion

### Summary of main findings

This systematic review and meta-analysis studied HF patient preferences regarding ADs, particularly focusing on surrogate decision-making, LSTs, and CPR. The pooled estimate revealed that 53.66% of patients preferred surrogate decision-making, increasing to 71% after excluding the outlier study by Formiga et al. (2004) [24]. For LSTs, 48.8% of participants indicated a preference to receive them, with the estimate increasing to 52.28% upon exclusion of Campos et al. (2022) [22]. In contrast, 43.32% of patients expressed a preference against receiving CPR, a proportion that differed with sensitivity analyses. These findings show a moderate tendency toward forgoing aggressive treatments and a substantial reliance on surrogate decision-makers, although with considerable heterogeneity across studies and populations.

Because EOL preferences are deeply shaped by cultural, social, and healthcare-system contexts, pooled estimates should be interpreted as broad indicators rather than universal values. The substantial heterogeneity observed across studies reflects meaningful contextual differences, and our meta-analytic approach was intended to summarize central tendencies while acknowledging this variation.

### Surrogate decision-making preferences

Preferences for surrogate decision-making (SDM) were present in over half of the patients studied, with this preference being particularly noticeable in Asian populations. Actually, ‘SDM’ refers to situations in which the surrogate makes decisions on behalf of the patient due to impaired decisional capacity. For instance, a majority of older Korean and Japanese adults preferred delegating medical decisions to family members rather than deciding independently [26–28]. While, Western populations had a greater inclination toward autonomy or shared decision-making [12, 13]. Interestingly, Cousino et al. (2023) [13] compared parents and young adults with heart failure and found that while 59% of patients wished to be the leading decision-makers, younger patients demonstrated a stronger preference for autonomy, whereas parents leaned toward shared decision-making or deferring to others.

Patients often choose spouses or adult children as surrogates [4, 25, 27]. However, surrogate designation does not always imply effective communication, as many studies emphasized that surrogates were often unaware of the patient’s actual treatment preferences [13]. This highlights the constant ethical debate between respect for autonomy and relational models of decision-making. Emotional burden, cultural values, and cognitive decline can complicate surrogate roles and emphasize the need of prompt and plain ACP discussions.

### Preferences Regarding Life-Sustaining Treatments (LSTs)

Preferences for LSTs were divided, with roughly half of the participants expressing interest in receiving such treatment. The types of LSTs assessed varied across studies and included mechanical ventilation, intubation, dialysis, and feeding tubes [12, 22, 27]. Patients in studies involving older or weaker populations [24, 29](e.g., Formiga et al., 2004; Trasancos-Escura et al., 2021) were more likely to reject LSTs, whereas those in more steady outpatient settings, such as Habal et al. (2011) [25], tended to accept them.

Importantly, treatment preferences were dynamic. Campos et al. (2022) [22] conducted a longitudinal study and reported considerable shifts in preferences over time, driven by changes in symptoms, emotional states, and family influence. These findings support the view that ACP should not be a one-time event, but rather a dynamic process that reflects patients’ changing values and clinical status.

Because the wording and framing of LST questions varied across studies, reported preferences should be interpreted within their specific clinical and cultural contexts.

### Cardiopulmonary Resuscitation (CPR) Refusal

The proportion of patients preferring to refuse CPR was substantial (43.32%) and varied significantly across studies. The highest refusal rate was reported by Campos et al. (2022) [22] at 87.6%, while Habal et al. (2011) [25] reported only 2%. Older age, functional decline, and poorer self-perceived quality of life were strongly associated with a preference against CPR [12, 29].

Remarkably, studies have demonstrated that patient preferences often change after physician-patient conversations about end-of-life care. In Young et al. (2017) [16], patients who had discussed end-of-life preferences were significantly more likely to decline CPR and understand hospice care. Similarly, Gordon et al. (2016) [4] found that patients who discussed prognosis with their physicians were more likely to have surrogate decision-makers and ACP documents in place. These results highlight the influence of informed discussions on CPR preferences.

We note that HF patients may view CPR as more potentially reversible due to the cardiac origin of their disease. This perception, along with cardiology practice patterns emphasizing rhythm correction, may influence CPR preferences and should be considered a potential confounder.

### Communication Gaps and Advance Care Planning (ACP) Barriers

Only 20.8% of patients included in Kim et al. (2020) [26] and 13.4% in Kim et al. (2021) [27] were not aware of ADs. Furthermore, Habal et al. (2011) [25] reported that 76% of patients were unaware of ADs. This low awareness is often reflected in AD completion rates, as well [28]. Despite a high proportion of patients expressing interest in ACP, discussions about EOL care and formal documentation were often lacking. In Kitakata et al. (2021) [28], only 6.6% had completed ACP documentation, and in Kim (2021) [27], only 13.3% had an AD in place. Even in countries with supportive policies, systemic inertia, prognostic uncertainty, and cultural taboos hamper ACP progress [13, 26].

Higher morbidity, manifested as frequent decompensations, symptom burden, and functional decline, may prompt earlier or more urgent ACP discussions. Patients experiencing repeated hospitalizations or worsening symptoms may be more receptive to discussing ADs, influencing both preferences and readiness for decision-making. Patients frequently mentioned emotional discomfort and lack of opportunity as barriers to communication [4, 13]. Cultural norms also played a considerable role. For instance, fatalistic beliefs and reluctance to discuss death were particularly prevalent among East Asian populations [26, 28], hindering open conversations about end-of-life preferences. These findings necessitate structured, culturally tailored ACP interventions involving both patients and families.

Knowledge and preference alignment could be significantly improved due to educational interventions. In the case of El-Jawahri et al. (2016) [23], Video-assisted interventions increased comfort care preferences (51% vs. 30%) and reduced preferences for CPR (68% vs. 35%) in comparison to verbal discussions. This type of intervention might be contributory in development of AD setting in other countries, as well. However, further interventional studies are required.

Cousino et al. (2023) [13] was the only study to examine Adolescents and Young Adults (AYAs) with advanced HF and reported noticeable discordance between patient and parents’ preferences. Whereas 45.3% of AYAs preferred active, patient-led decision-making, parents preferred shared decision-making. These findings underscore the value of family-centered, age-specific communication methods. Moreover, data for other range of ages may not be generalizable for AYA populations. Limited ACP awareness in Kim et al. (2020) may reflect cultural norms discouraging open discussion of EOL issues, suggesting that cultural factors may be a primary driver rather than communication gaps alone.

### Cultural influences on advance directive preferences

This review focused on the substantial influence of cultural context on patient preferences regarding advance directives, especially in areas such as surrogate decision-making, acceptance of life-sustaining treatments, and CPR. Studies conducted in East Asian countries such as Korea and Japan constantly demonstrated a convincing preference for family-centered or surrogate decision-making over autonomous patient decision-making [26–28]. These preferences are deeply rooted in traditional cultural norms that prioritize family harmony, filial responsibility, and deference to authority in healthcare decisions.

In contrast, studies conducted in Western countries—such as the United States, Canada, and several European nations—reported more diverse patterns. While some patients preferred shared decision-making, many leaned toward personal autonomy and wished to make treatment decisions themselves [4, 12, 13]. Even within Western populations, variability was apparent across generations. Cousino et al. (2023) [13] found that younger adults with heart failure more strongly valued independent decision-making compared to their parents, suggesting that cultural attitudes toward autonomy may be evolving.

Cultural context also appeared to affect acceptance of LSTs and CPR. For instance, Spanish patients [24, 29] showed a greater tendency to forgo aggressive interventions such as CPR and only benefit from palliative and even home care than those in North American studies, probably reflecting different views on the quality of life, death, and spiritual beliefs. Also, Spanish studies showed low awareness of AD in their population and emphasized on barriers to discuss about CPR and AD [24, 29]. Whereas, patients in Canadian and Japanese contexts [25, 28] showed greater openness to receiving CPR or mechanical support, potentially due to more institutionalized norms around life-prolonging care or discomfort with initiating end-of-life debates.

Preference regarding starting ACP discussions differed by regions. Japanese patients preferred the initiation of ACP during repeated admissions [28], whereas patients’ engagement in American sample was low during hospitalization [16].

To conclude, these findings highlight that ACP is not culturally neutral. Preferences for autonomy, surrogate involvement, and aggressive treatment vary meaningfully between regions, ethnicities, and belief systems. Hence, clinicians must be sensitive to these cultural differences to avoid one-size-fits-all approaches to advance care planning. Modifying conversations to reflect patients’ cultural values and social expectations may improve the effectiveness, receptivity, and ethical soundness of ACP interventions.

We observed that ACP engagement varied by clinical setting. Outpatients demonstrated greater prior exposure to ACP, while inpatients, particularly those hospitalized for acute HF decompensation, expressed higher willingness but lower preparedness for ACP discussions. Tertiary-care centers showed higher ACP documentation rates than community hospitals, suggesting that institutional resources and clinician familiarity may influence ACP uptake.

### Clinical and research implications

Findings from this review have numerous clinical implications. First, healthcare providers should initiate ACP conversations early, especially after major clinical events such as hospitalization or functional decline. These conversations should be repeated and tailored to the patient’s changing condition and cultural background. Second, ACP should move beyond general preferences and explore specific decisions regarding treatments such as CPR, mechanical ventilation, and dialysis. Finally, clinicians should explore patients’ decision-making preferences and help patients communicate their wishes clearly with both healthcare proxies and family members. Future research should further examine the longitudinal evolution of preferences and explore intervention models (e.g., nurse-led programs, decision aids) that can enhance ACP engagement across diverse populations.

### Strengths and limitations

This systematic review and meta-analysis synthesized findings from diverse studies to provide a comprehensive overview on HF patients’ preferences for ADs and LSTs. However, numerous strengths and limitations should be acknowledged: The review captured a wide range of geographic, cultural, and demographic contexts, offering insights into how cultural and regional differences influence ACP and AD preferences. Studies that evaluated interventions, such as video decision aids or ACP education, provided novel insights into how knowledge and decision-making preferences can be influenced and improved. Definitions of ADs, as well as preferences and choices for LSTs, varied widely across studies. This heterogeneity complicated the synthesis of findings and posed challenges for meta-analysis. Variations in the reporting of outcomes and methodologies across studies made it difficult to directly compare results. For example, the preference for CPR or other LSTs was sometimes framed as a binary choice, while other studies presented nuanced data on developing preferences. The heterogeneity in study design, population characteristics, and cultural contexts limits the generalizability of findings. Preferences observed in one cultural or clinical setting may not fully apply to others.

We caution that pooled estimates reflect aggregated patterns across diverse cultural and healthcare contexts and should not be interpreted as uniform preferences. Contextual variation remains substantial and clinically meaningful, and future research should examine cultural and system-level moderators of EOL decision-making.

## Conclusion

Preferences surrounding advance directives, SDM, LSTs, and CPR are highly diverse and context dependent. They are shaped by individual experiences, evolving health status, and deeply rooted cultural and familial values. The findings from this review suggest that it is neither feasible nor appropriate to adopt a universal approach to EOL care planning. Instead, clinicians must prioritize culturally sensitive, person-centered conversations that accommodate shifting preferences over time and across circumstances. Recognizing and respecting this diversity is central to ethical and effective advance care planning. Customized interventions like video-assisted decision aids and repeated ACP conversations are particularly essential to tailor care matching better with patient values, which may also change over time. Future research should address knowledge gaps, dynamic preferences, and the needs of specific subgroup populations, such as AYAs and culturally diverse groups. Understanding and respecting patient preferences is essential for enhancing EOL care, ensuring that healthcare aligns with both clinical guidelines and the values of patients. By prioritizing patient-centered approaches, we can create a more compassionate healthcare environment that empowers patients to make informed choices reflective of their unique experiences at the EOL. Finally, Mixed-methods reviews could integrate qualitative perspectives to enrich understanding of how HF patients articulate values, negotiate family roles, and interpret LSD, LSTs and CPR decisions.

## Data Availability

Data are available through all figures and tables in the manuscript and are all disclosed.
